# SUSTAINABILITY OF CARE COORDINATION FOR PEOPLE WITH DEMENTIA WHO LIVE ALONE: LESSONS FROM A FEDERAL GRANT PROGRAM

**DOI:** 10.1093/geroni/igad104.3612

**Published:** 2023-12-21

**Authors:** Yoon Chung Kim, Michael Lepore, Kate Gordon, Heather Menne

**Affiliations:** University of Maryland Baltimore, Baltimore, Maryland, United States; University of Maryland School of Nursing, Baltimore, Maryland, United States; Splaine Consulting, Columbia, Maryland, United States; Miami University, Oxford, Ohio, United States

## Abstract

Approximately one-third of older adults with dementia or cognitive impairment in the US live alone. People living alone with dementia (PLAWD) are at high risk for undesirable outcomes including malnutrition and falls, and the absence of cohabitants—who typically coordinate care—can contribute to unnecessary hospitalization and institutionalization. Since 2014, the Administration for Community Living (ACL) within the US Department of Health and Human Services has required PLAWD-focused services in ACL’s Alzheimer’s Disease Program Initiative. As part of a larger study of PLAWD-focused service sustainability, we examined ACL project final reports (n=73) to describe care coordination services for PLAWD and their sustainability plans. We excluded projects without services for PLAWD (n=14) or care coordination services (n=29) and where the lead organization closed (n=2) or project director died (n=1). Of 27 project reports indicating PLAWD-focused services and care coordination services, most were community-based organizations (CBOs, 67%); others were health systems/medical centers (15%), universities (11%), and government agencies (7%). PLAWD-focused care coordination services commonly entailed identifying PLAWD, assessing their needs, developing care plans, connecting PLAWD to need-relevant services, and purchasing supportive equipment, and sometimes involved training volunteers. Most organizations (70%) planned to sustain PLAWD services. The primary sustainability barrier faced by CBOs was lack of funding, whereas for health systems and universities partnering with CBOs was key to sustainability. This review of ACL-supported care coordination services for PLAWD and their sustainability highlights the need for research on the effectiveness of care coordination services for PLAWD and of different approaches to service sustainability.

